# Tables of Toxicity of Botulinum and Tetanus Neurotoxins

**DOI:** 10.3390/toxins11120686

**Published:** 2019-11-22

**Authors:** Ornella Rossetto, Cesare Montecucco

**Affiliations:** 1 Department of Biomedical Sciences, University of Padova, Via Ugo Bassi n. 58/B, 35121 Padova, Italy; 2 Institute for Neuroscience, National Research Council, Via Ugo Bassi n. 58/B, 35121 Padova, Italy; cesare.montecucco@gmail.com

**Keywords:** botulinum neurotoxin, tetanus neurotoxin, botulism, tetanus, toxicity, lethal dose

## Abstract

Tetanus and botulinum neurotoxins are the most poisonous substances known, so much so as to be considered for a possible terrorist use. At the same time, botulinum neurotoxin type A1 is successfully used to treat a variety of human syndromes characterized by hyperactive cholinergic nerve terminals. The extreme toxicity of these neurotoxins is due to their neurospecificity and to their metalloprotease activity, which results in the deadly paralysis of tetanus and botulism. Recently, many novel botulinum neurotoxins and some botulinum-like toxins have been discovered. This large number of toxins differs in terms of toxicity and biological activity, providing a potential goldmine for novel therapeutics and for new molecular tools to dissect vesicular trafficking, fusion, and exocytosis. The scattered data on toxicity present in the literature require a systematic organization to be usable by scientists and clinicians. We have assembled here the data available in the literature on the toxicity of these toxins in different animal species. The internal comparison of these data provides insights on the biological activity of these toxins.

## 1. Introduction

Microorganisms produce hundreds and hundreds of different protein toxins that act with different mechanisms to subvert the defense systems and physiology of their hosts (vertebrates, invertebrates, and plants) to the advantage of the toxigenic species. The toxicity of bacterial toxins acting on vertebrates has been traditionally tested in laboratory animals, and most of the recent data are determined in selected mice strains. These in vivo methods are progressively substituted by in vitro methods, which, however, have not reached the physiological complexity of the animal hosts where these toxins act to cause disease. 

The last table of toxicity of bacterial toxins in vertebrate animals was published long ago [1] and never revised afterwards. Meanwhile, the literature on bacterial toxins has grown enormously, and it is very difficult to elaborate a single paper containing the toxicity data of all known bacterial protein toxins. Moreover, new methods of toxin purification have been developed, leading to the production of purer toxins with higher specific activity. We reasoned that it was time to assemble comprehensive tables of toxicity for tetanus (TeNT) and botulinum neurotoxins (BoNTs) to provide an updated set of data and to contribute to further research in the field, as well as to their use in animal therapy. Here, we present tables of toxicity for TeNT and for the many BoNTs in mice, and in all other animals for which toxicity data are available. These tables include values obtained with different ways of inoculation and in different organisms.

### 1.1. Clostridial Neurotoxins

Tetanus and botulinum neurotoxins are produced by different species of anaerobic bacteria of the genus *Clostridium* [2,3]. They cause the neuroparalytic syndromes of tetanus and botulism in mammals and other vertebrates [4,5]. They are the most toxic substances known. Because of these characteristics and the lack of prevention of botulism by vaccination, BoNTs are included in the category A list of agents that could be used in bioterrorism [6,7].

One serotype of TeNT is known, whilst eight different serotypes of BoNT have been characterized (BoNT/A, /B, /C, /D, /E, /F, /G, and /X) [2,8]. TeNT proteins with little variation in amino acid sequence, that do not alter significantly their antigenicity, have been recently reported [9,10], whereas many dozens of BoNTs with very variable primary structures and ensuing different biological properties and antigenicity have been described. They are defined as subtypes of a given serotype and indicated with arabic numbers (for example BoNT/A1, BoNT/A2, etc.) [11]. In addition, chimeric BoNTs are known: BoNT/CD, BoNT/DC, BoNT/FA [3].

### 1.2. Structure of Clostridial Neurotoxins and Their Complexes

Despite the existence of a high number of isoforms, all BoNTs and TeNT are structurally similar and consist of two chains linked by a unique interchain disulphide bond: A light chain (L; 50 kDa) and a heavy chain (H; 100 kDa). In the case of BoNTs, the 150 kDa holotoxin is produced by bacteria, together with nontoxic accessory proteins, to form high molecular weight progenitor complexes (PTCs) of various sizes [2]. Different variants of a 140 kDa non-toxic, non-hemagglutinin protein (NTNHA) form a 1:1 complex with BoNT dubbed M-PTC (BoNT:NTNH, ~300 kDa or 12S). In the M-PTC complex, the two proteins shield each other against acidic pH and proteolytic degradation. In addition, various combinations of hemagglutinin proteins (HAs) associate to M-PTC to form the L-PTC (BoNT:NTNH:HA1:HA2:HA3, ~500–760 kDa or 16S), and LL-PTC (dimer of L-PTC, ~900 kDa or 19S) complexes. The BoNT/A toxins may be complexed in all three forms (LL-PTC, L-PTC, and M-PTC) and BoNT/B, /C, /D, and /G in two forms (L-PTC and M-PTC). In contrast, bacterial serotypes /E and /F do not have the HA genes and may only produce BoNT complexed in the M-PTC form. The role of PTC in assisting BoNT to breach the intestinal barrier has been recently elucidated in molecular details, explaining the much higher oral toxicity of the PTC with respect to the free BoNT (see below) [12,13].

### 1.3. Toxicity Values for Botulinum Neurotoxins and Their Limitations

Owing to its neurospecificity, long duration of neuroparalysis, reversibility, and limited diffusion of paralysis after injection, BoNT/A1 has become a therapeutic of choice for all those human syndromes and physiological alterations caused by hyperfunction of peripheral cholinergic nerve terminals [14,15,16]. The amount of BoNT/A1 to be injected is estimated in International Units, which correspond to the mouse lethal dose 50% (MLD50). Both PTC and toxin alone BoNT/A1 formulations are commercially available for clinical use [16].

It should be considered that toxicity figures may suffer from limitations derived from multiple factors: (1) toxins of different purity were used in different reports, particularly in the past, when methods to define the chemical and physical homogeneity of a protein preparation were not standardized; (2) in older papers, toxicity was expressed as the minimal lethal dose, that is defined as the minimal dose of toxin capable of killing the injected animal, but there is variation among different experimenters about the number of animals to be used and of time to death [17]; (3) amounts of protein toxins were, in the past, frequently measured not in units of weight of the toxin, but, rather, in flocculating units or in milligrams of nitrogen. This latter figure has been translated here in units of weight with the formula: LD50 expressed in milligrams of nitrogen (N) × 6.25 considering that the nitrogen content of pure clostridial neurotoxins is about 16%; (4) different strains of one animal species were used in different laboratories; and (5) figures for the different routes of injection and the different housing temperatures for cold-blooded animals are given here, but subtle differences among laboratories cannot be estimated, particularly for intracranial or intraspinal values. However, in many cases, we report toxicities as ranges or intervals that summarize data from different reports, and this attenuates the limitations listed above. As the portal of entry of the toxin determines the value of toxicity, we have differentiated the data in terms of the different routes of administration. Most data derive from experiments performed in the laboratory using mice, but it is well known that toxicity of a given toxin strongly depends on the animal species, and this is reported here, whenever possible.

Another aspect that deserves attention and it is difficult to be evaluated, is that the toxicity recorded in the laboratory is always lower than the corresponding one occurring in wildlife. Indeed, even minimal functional deficits caused by very small doses of the neurotoxins that can be overcome, particularly for BoNTs, with a total recovery of a normal physiological state in the animal house, are lethal in the environment. Consider, for example, that even minor visual deficits, which are the first evident symptoms of botulism, do not affect survival in a cage in the animal house, whilst are lethal in the wilderness. Indeed, botulism is a rare disease in humans, whilst it is a major killer in wildlife. What matters in the wilderness is the amount of toxin sufficient to cause a minimal loss of physiological performance of the intoxicated animal incompatible with life (i.e., the capability of performing flying for birds, or to govern movements among fish) or the minimal dose that makes the intoxicated animal incapable to escape predators. A final introductory point is to be dedicated to the so-called time-to-death assay introduced to save time in testing the activity of BoNTs [18]. This method employs doses of several orders of magnitude higher than the LD50 in order to cause animal death within few tens of minutes. As discussed elsewhere [16], such high doses of BoNTs may bind neurons, tissues, and organs that are not involved in death by botulism. When data derived from this assay are reported, it is indicated at the bottom of the table.

## 2. Botulinum Neurotoxins

### 2.1. Mouse Lethal Dose 50%

Table 1 reports the mouse lethal dose 50 values (LD50) of different BoNTs in *Mus musculus*, as the dose that causes 50% death in 20 gr caged mice within 4 days following intraperitoneal injection. 

The toxicity values are reported as ranges between the lowest and the highest LD50 found in literature, with the omission of many values obtained before modern methods of protein purification and analysis were available. The current purification protocols deliver neurotoxins with high specific toxicities, from 10^7^ to 10^8^ MLD50s/mg of proteins, where the unit of weight refers to the purified 150 kDa di-chain toxin. Absolute measurements, such as LD50 data, are not biological constants, as they are highly dependent on the assay conditions. Indeed, lethality assessed in the laboratory may be influenced by the mice strain and specific conditions used, including cage density, time of injection during the day, and diet, that are not usually reported. More importantly, the effect of mixing purified BoNT or TeNT with gelatin or purified albumin or colloids was found long ago to increase their potency, stability, and reproducibility after the dilution procedures necessary to measure toxicity [19,20,21,22]. Possibly, these so-called “carriers” prevent self-aggregation or attachment of the diluted proteins to plastics or glass. All LD50 reported in Table 1 have been determined by mouse bioassay, in which 0.2% gelatin was present in the dilution buffer.

### 2.2. Toxicity in Different Animal Species

Table 2 provides a comparative estimation of the lethal doses of BoNTs following parenteral delivery, expressed for the species for which data are available as per unit of body weight with respect to those found in mice; this is an extension of the picture reported in [47]. Toxicology data are available only for a limited number of the many BoNTs so far identified and only for few vertebrate species. A further limitation is provided by the fact that available toxicological studies were performed with BoNT preparations of different purity and storage conditions, and, more importantly, by different routes of administration. Consequently, the estimates of the relative toxicity of BoNTs in different animal species are approximate. However, the general picture points out that mice, guinea pigs, rabbits, and monkeys are very sensitive to all the toxin types, with the exception of BoNT/D for monkeys. The low toxicity of BoNT/D in monkeys correlates with the recent experimental electrophysiology study in humans that showed BoNT/D to be poorly effective in inducing local paralysis after injection into the extensor digitalis brevis muscle [42]. Horses are not present in the table, but epidemiological data indicate that they are BoNT-sensitive animals.

With the current knowledge of the protein receptors and the cleavage sites of the SNARE proteins by each BoNTs, the resistance/sensitivity of an animal species to a specific toxin can be explained in many cases at the molecular level. For example amino acid substitutions in VAMP1 at the cleavage site of BoNT/B in rat and chicken and of BoNT/D in primates may explain the low sensitivity of these animal species to the respective BoNTs [48,49].

### 2.3. Oral Toxicity in Mice

The available data on the intravenous (i.v.), the intraperitoneal (i.p.), and the intramuscular (i.m.) routes of BoNTs injection indicate that toxicity is very similar for these three modes of administration. However, since the most frequent form of botulism is food poisoning, the oral route of administration resembles better than the parenteral one the natural intoxication. Indeed, the oral toxicity of the different BoNTs have been investigated mainly in mice. Table 3 reports the oral LD50 of BoNT/A to BoNT/F in relation to their molecular complex organization. These data show that the oral LD50 value is from a thousand to a million times larger than that caused by intraperitoneal injection, indicating that the process of animal intoxication is rather inefficient by the oral route. Moreover, the table highlights the fact that the free BoNT is sensitive to inactivation by the acidic environment and digestive proteases present in the gastrointestinal (GI) tract [13]. In fact, the PTCs of the different serotypes exhibit a much higher oral toxicity than the corresponding free BoNTs [63,64,65] (Table 3). Among the toxins tested, BoNT/B L-PTC showed an extraordinarily high oral toxicity in mice [35,63], and this is particularly evident for BoNT/B complex from Okra strain, which is the most potent in oral toxicity and correlates with its high fatality rate in causing human botulism [64]. Such a high oral toxicity of BoNT/B L-PTC was also observed in monkeys [66]. The oral toxicities of BoNT/C L-PTC and BoNT/D L-PTC toxins were also high, being close to that of BoNT/B L-PTC toxin. BoNT/A and /B are the major cause of human botulism, while BoNT/C and /D predominantly cause botulism in cattle, poultry, and wild birds. The different host susceptibility observed among different BoNT serotypes could be partly caused by the different intestinal absorption of the progenitor toxin. The amount of BoNT/C L-PTC necessary to cause botulism via the oral route is 100-fold than that by the intravenous route in the case of geese [67], and more than 5000-fold in mice [44], indicating that the intestinal absorption of BoNT/C progenitor toxin in birds is more efficient than in mice. Structural studies on HA33/A, /B, and /C have revealed serotype-specific HA33–glycan interactions [12,68,69], suggesting that this biochemical interaction may contribute to the host tropism of the different BoNT serotypes. Another factor likely to play a role in determining this outcome is the sensitivity of the different BoNT complexes to the proteases present in the lumen of the GI tract. Accordingly, no difference in toxicity was observed for the 150-kDa holotoxin, and the PTC complex when administered by i.p. [35,70], a route which ensures disassembly of complexes in vivo at the mildly alkaline physiological pH of blood and by the HA proteins binding to blood components [71,72].

### 2.4. Toxicity of BoNT/A1 in Human and Therapeutic Doses

Botulinum toxins are, at the same time, the causative agents of foodborne botulism, potential attraction for terroristic and military misuse, and pharmaceutical agents currently approved for the treatment of dozens of neurological and non-neurological human diseases and for cosmetics. Therefore, considering the different aspects of health and consumer protection, security, as well as the medical use, the determination of potency of BoNTs in humans is of paramount importance. The toxicity of BoNT/A1 L-PTC in humans was estimated by extrapolation from primate studies to be 1 µg/kg of body weight when taken orally, 10 ng/kg by inhalation, and 1 ng/kg intravenously or intramuscularly [6]. The potency of the different BoNT/A1 preparations commercially available for medical/esthetic uses is expressed as Units (U), where 1 U corresponds to 1 LD50 in the mouse bioassay [16]. The injected dose of BoNT/A1 in humans varies from 20 U for the treatment of glabellar lines [73] to 800 U for spasticity [74]. Considering that 1 U of BoNT/A1-based drug corresponds to few picograms of toxin, it turns out that even the maximal clinical dose used is about 20-fold lower than the intramuscular LD50.

## 3. Tetanus Neurotoxin

### 3.1. Toxicity in Different Mammals

Table 4 reports the data of toxicity of TeNT in mammals, expressed as multiple of the mouse minimal lethal dose (MMLD). The choice of this parameter is due to the fact that the more accurate mouse lethal dose 50% (MLD 50%) is rarely available for this neurotoxin. Given the well-known fact that different toxicities are associated with different TeNT preparations [75], we report here the highest toxicity figure among those available in the literature, considering the intrinsic activity of the toxin preparation as the major source of variation. This table does not include a common experimental animal such as the rat, as this animal species is rather resistant to TeNT. Indeed 100–6000 MMLD was used to study the development of local tetanus in rats [76]. Rat resistance was attributed to a mutation at the site of TeNT cleavage of VAMP1, which is also present in chickens, another tetanus resistant animal species (see Table 5) [48]. It is long known that tetanus results from an action of TeNT in the spinal cord [77,78,79], and the finding that TeNT injected into sciatic nerve was more toxic indicated that it is transported from the periphery to the spinal cord via intraneural transport [47,80,81]. Accordingly, the figure resulting from the intra-spinal cord injection is even lower, particularly so in cats and dogs, indicating that in these animals, the retroaxonal transport of TeNT is less efficient than in other animals [82].

Tetanus toxin is most potent when introduced into the central nervous system, and least potent by mouth; the toxicity by oral route being 1/200,000–1/1,200,000 that of the parenteral route [83].

### 3.2. Toxicity in Non-Mammal Animals

Table 5 shows the toxicity values obtained with three species of domestic birds, which show the common properties of being very resistant to TeNT. Lizards and other reptiles have been studied by [87], but the number of animals available was small and the statistics of these studies were, by necessity, not adequate. However, a general finding was that cold-blooded animals develop tetanus after the injection of TeNT if kept at room temperature or higher, but not in the cold. Summarizing these results, lizards (genuses: *Dipsosaurus*, *Lacerta*, *Pachydactylus*, *Pseudocrdylus*, *Zonurus*) were found to be sensitive to TeNT at doses comparable to the minimum lethal dose of guinea pigs and only when kept warm. Chameleon and crocodile required >100 times more TeNT, whilst tortoises were found to be almost insensitive to TeNT, as a thousand times the lethal doses of guinea pigs were required to cause tetanus. A viperidae South African snake was found to develop tetanus with doses 10^4^ higher than those required in guinea pigs, whilst colubridae snakes can be considered resistant to TeNT, as massive doses were necessary to cause tetanus in animals kept at 37 °C.

A final comment on the effect of TeNT in frogs, which are resistant to tetanus in the cold. As other poikilothermic animals, they develop tetanus in a temperature-dependent way. Symptoms appear faster the higher is the environmental temperature with the animals surviving if kept in the cold [88]. This is fully in agreement with the fact that endocytosis is practically absent below 15 °C, and that the L chain of TeNT is a metalloprotease whose enzymatic activity is temperature-dependent.

## 4. Conclusions

Clostridial neurotoxins are the most potent toxins, and their extraordinary toxicity in different animal species has been long known, especially from studies performed in the past century. They are of special interest because TeNT is still a major disease in some countries, and BoNT/A1 has become a major human therapeutics. The last review of clostridial neurotoxin lethal doses derived from studies dating back more than 40 years ago [1]. However, in the last decades, the new protocols for toxin purification lead to purer toxins, which have an even higher neurotoxicity than in the past. Moreover, many novel BoNTs have been identified recently, and BoNTs can be produced as recombinant proteins in *Escherichia coli*, calling for the present updated and extended review on the toxicity of clostridial neurotoxins. In some cases, recombinant BoNTs were found to be more potent than their clostridial counterparts [90,91]. The more striking example is BoNT/D, whose mouse LD50 was found to be as low as 0.02 ng/Kg [28], which corresponds to about 40,000 molecules per mice. 

The extremely high potency of clostridial neurotoxins is due to the unique combination of two factors: (a) the very rapid binding to neurons, whose integrity is essential for survival, and (b) their enzymatic activity highly specific for the three SNARE proteins, whose cleavage is sufficient to block neurotransmitter release with ensuing neuroparalysis [2,3]. This is evident for vertebrates, but it is also true for invertebrates—particularly for flying insects, as shown by the very recent discovery of a BoNT-like neurotoxin specific for *Anopheles* mosquitos [92].

This gathering of toxicity data is predicted to be a useful starting point to address further studies of the molecular and cellular basis of the action of TeNT and BoNTs in different animal species, which may fill relevant gaps in our present knowledge of the pathogenesis of tetanus and botulism.

## Figures and Tables

**Table 1:** Intraperitoneal (i.p.) lethal dose 50 values (LD50) of botulinum neurotoxins (BoNTs) in mice.

BoNT Type.	i.p. LD50 (ng/Kg)
A1	(0.25–0.45) [23,24,25,26,27,28,29,30]
A2	(0.11–0.53) [24,26,27,31]
A3	0.85 [27,32]
A4	(400–500) [27]
A5	(0.35–0.40) [27,33]
A6	(0.26–0.3) [30,34]
B1	(0.21–0.50) [28,34,35,36,37]
B2	0.4 [38]
C1	(0.92–2.3) [39,40,41]
C/D	(0.8–1.92) [39,41]
D1	(0.02–0.83) * [28,29,39,41,42]
D/C	0.05 [41]
E1	(0.65–0.84) [24,43]
E3	3.05 [37];
F1	(2.4–3.6) ^#^ [37,44]
FA	(1.30–2.2) [38,45]
G	5.00 [46]

The toxicity values are reported as ranges between the lowest and the highest LD50 found in the literature. For each range, references that report values within the range are cited. * recombinant toxin in [28]; ^#^ BoNT/F in [44] is in M-PTC form; the i.p. LD50 is extrapolated from the time-to-death assay in [39,41,44].

**Table 2:** Comparative toxicity of BoNTs in different animals expressed as multiple of the mouse LD50/Kg.

BoNT Type	Source	Mouse	Rat	Guinea Pig	Rabbit	Dog	Cat	Monkey	Fowl	Pigeon	Turkey	Zebra Fish *
A	[50]	1	2.5									
	[51]	1		0.5	0.3				15			
	[52]	1			0.8 (i.m.)							
	[53]	1						0.78 (i.m.)				
	[54]	1						0.5 (i.v.)				
	[55]	1						11 (inh.)				
	[56]	1										100 (ic.)
B	[50]	1	1000									
	[52]	1			0.1 (i.m.)							
	[55]	1						432 (inh.)				
	[57]	1						150 (inh.)				
	[58]	1		0.2								
	[59]	1		0.3								
C	[60]	1	6	1	0.1	1.000	800	0.3	2000	20		
	[61]	1									7 (i.v.)	
	[56]	1										400 (ic.)
D	[60]	1	320	0.2	0.2	100.000	15.000	100	100.000	2000		
	[56]	1										20 (ic.)
E	[60]	1	40	0.5	1	100	400	1	25	25		
F	[62]	1						0.5 (s.c.)				

* IC50 immobilizing dose intracelomatic injection; i.m., intramuscular; i.v., intravenous; s.c., subcutaneous; inh., inhalation; ic., intracelomatic.

**Table 3:** Oral toxicity of BoNTs of different molecular size in mice.

BoNT Type	Molecular Form of Toxin	Oral LD50 ^a^	Relative Oral Toxicity ^b^
A	LL-PTC	0.12 × 10^6^	358
	L-PTC	2.2 × 10^6^	19.5
	M-PTC	3.6 × 10^6^	11.9
	Holotoxin	43 × 10^6^	1
B	L-PTC	1.5 × 10^3^	28,700
	M-PTC	1.1 × 10^6^	39
	Holotoxin	24 × 10^6^	1.8
C	L-PTC	5.3 × 10^3^	8113
	M-PTC	1.6 × 10^5^	268
D	L-PTC	6.2 × 10^4^	693
	M-PTC	3.7 × 10^5^	116
E	M-PTC	3.7 × 10^5^	116
F	M-PTC	1.1 × 10^6^	39

^a^ Equivalent number of i.p. LD50 (determined by the time-to-death assay). ^b^ The oral toxicity of type A holotoxin was taken as 1. Data for BoNT/A, /B, and/F are from [63] and for BoNT/C, /D, and /E from [44].

**Table 4:** Minimal mouse lethal doses of tetanus toxin in ng/kg in different mammals and different routes of administration ^a^.

Way of Inoculation	Mouse	Guinea Pig	Rabbit	Cat	Dog	Goat	Sheep	Horse	Monkey	Human
intramuscular (im)	0.15	0.2	3	600	150	0.24	0.4	0.2	0.4	0.2 ^b^
intravenous (iv)		0.2	12	480	240				2	
intraperitoneal (ip)	0.15									
subcutaneous (sc)		0.2	12							
intraventricular		0.2	12							
intra-sciatic nerve			1.2						0.25	
intra-spinal cord			0.12	2.0	0.1					
intra-medulla			0.012		0.15					
intra-ventriculum		0.1–0.2	1.2							

^a^ Adapted from [17,47,81,84,85,86]; data have been converted into nanograms/kilograms for a better comparison. ^b^ TeNT toxicity value for humans is the results of extrapolation from monkey data [6] and from records of accidental inoculations quoted by [47].

**Table 5:** Toxicity of tetanus toxin expressed as multiple of the mouse minimal lethal dose (MMLD).

Way of Injection	Hen	Pigeon	Goose	Frog	Gold Fish	Lizard
**i.m.**	100,000	12,000	3000	3000	17	2
intra-brain *						2

Data from [47,87,88,89]. * No information was given on the exact point of injection within the brain.
